# Phosphorylation regulates the subcellular localization of Cucumber Mosaic Virus 2b protein

**DOI:** 10.1038/s41598-017-13870-7

**Published:** 2017-10-18

**Authors:** Katalin Nemes, Ákos Gellért, Asztéria Almási, Pál Vági, Réka Sáray, Katalin Kádár, Katalin Salánki

**Affiliations:** 1 Plant Protection Institute, Centre for Agricultural Research, Hungarian Academy of Sciences, Budapest, Hungary; 2 Institute for Veterinary Medical Research, Centre for Agricultural Research, Hungarian Academy of Sciences, Budapest, Hungary; 3 Department of Plant Anatomy, Eötvös Loránd University, Faculty of Sciences, Budapest, Hungary

## Abstract

The 2b protein of *Cucumber mosaic virus* has a role in nearly all steps of the viral cycle including cell-to-cell movement, symptom induction and suppression of antiviral RNA silencing. Previous studies demonstrated the presence of 2b protein in the nucleus and in cytoplasm as well. Phosphorylation site of 2b protein is conserved in all CMV isolates, including proposed constitute motifs for casein kinase II and cyclin-dependent kinase 2. To discern the impact of 2b protein phosphorylation, we created eight different mutants to mimic the non-phosporylated (serine to alanine) as well as the phosphorylated state (serine to aspartic acid) of the protein. We compared these mutants to the wild-type (Rs-CMV) virus in terms of symptom induction, gene silencing suppressor activity as well as in cellular localization. Here, in this study we confirmed the phosphorylation of 2b protein *in vivo*, both in infected *N. benthamiana* and in infiltrated patches. Mutants containing aspartic acid in the phosphorylation site accumulated only in the cytoplasm indicating that phosphorylated 2b protein could not enter the nucleus. We identified a conserved dual phosphorylation switch in CMV 2b protein, which equilibrates the shuttling of the 2b protein between the nucleus and the cytoplasm, and regulates the suppressor activity of the 2b protein.

## Introduction

Posttranslational protein modification is a common mechanism for regulation of protein function in the plant and animal kingdom and can alter several aspects of protein function. It can modulate the biological activity of a protein, the interactions with other proteins, signal transduction, regulation of transcription, survival and apoptosis, affect the translocation of a protein from one subcellular compartment to another, can modify the half-life of the protein, or alter the conformation of a protein leading to switching the activity of a protein on or off^1^. Two main types of post translational modification exist: the cleavage of a protein backbone or the covalent modification of a protein. These events are enzyme-catalysed processes. The main types of the covalent modifications are phosphorylation, acylation, alkylation, glycosylation and oxidation of a certain amino acid side chain. Among these modifications phosphorylation is presumed to be the most frequent modification^2^, 30% of proteins in eukaryotic cells are phosphorylated. Several protein kinases in the plant kingdom have been functionally identified. They transfer phosphate groups from ATP to serine, threonine or tyrosine, and this process is reversible. The mitogen activated protein kinases (MAPKs) and calcium-dependent protein kinases are conserved and are involved in resistance to plant viruses and stress responses^3,4^. Other protein kinases were referred to as resistance (R) gene products^5,6^.

The currently available experimental results clearly demonstrate that protein phosphorylation should have a fundamental role in the regulation of viral infection, but in the case of plant viruses only limited data are available. The phosphorylation of the polymerase subunit of the replicase complex was proved in the case of *Cucumber necrosis virus* (CNV) and *Cucumber mosaic virus* (CMV)^7,8^. In these cases, phosphorylation can inhibit the binding of the viral RNA and could serve as an on/off switch in viral RNA binding and release. The phosphorylation of *Potato virus* A (PVA) and *Turnip crinkle virus* (TCV) replicase proteins were also reported. In these cases the fine tuning of viral RNA synthesis was regulated by phosphorylation. The phosphorylation of the movement protein (MP) of *Tobacco mosaic virus* (TMV)^9,10^, *Potato leafroll virus* (PLRV), *Abutilon mosaic virus* (AbMV) and CMV were also reported. The MP of TMV is phosphorylated by a plasmodesmal-associated protein kinase (PAPK)^11^ and the phosphorylation blocks RNA traffic through the plasmodesmata. In the case of PLRV the plasmodesmata targeting of the MP is influenced by phosphorylation^12^, the AbMV MP phosphorylation affects symptom development and viral RNA accumulation^13^ and phosphorylation of TGB1 of *Barley stripe mosaic virus* (BSMV) promotes its movement in monocots and dicots^14^. One more protein, the capsid protein phosphorylation was also reported. The PVA capsid protein is phosphorylated by the protein kinase CK2α, and phosphorylation inhibited the binding of PVA CP to RNA, so it plays an important regulatory role in virus infection^15^. In the case of *Bamboo mosaic virus* (BaMV) the phosphorylation of the coat protein (CP) regulates cell-to-cell movement through modulating RNA binding^16^. The phosphorylation of *Groundnut bud necrosis virus* (GBNV) nucleocapsid protein was also reported, but the function is still in question^17^.

CMV is one of the most important plant viruses infecting more than 1200 plant species including monocots and dicots as well. The genome of CMV consists of three positive stranded RNAs, encoding five proteins (1a, 2a, 2b, MP and CP). Previously the phosphorylation of CMV proteins was reported in two cases. The phosphorylation of the polymerase subunit of the replicase complex was described *in vivo* and its regulatory role in the formation of the replicase complex was proved^8^ and the phosphorylation of MP was reported in transgenic tobacco plants, but it was not examined in virus infected plants in details^18^.

The CMV 2b protein is multifunctional; it has a role almost in all steps of the viral cycle. It is one of the first described suppressors of antiviral RNA silencing, recognizing and binding not only RNA (siRNA, miRNA) but the protein components (AGO1 and AGO4) of the defense machinery as well^19,20^. It has also roles in symptom induction, evasion of the defense mechanism mediated by salicylic and jasmonic acid^21,22^ as well as in cell-to-cell movement^23^. Cell fractionation studies suggested earlier that during viral infection CMV 2b protein accumulated mainly within the cell nucleus, apparently in insoluble form^24^; tagged 2b protein with reporter markers confirmed its predominant nuclear localization in a CMV-free environment^25,26^. Though, subsequent studies demonstrated the presence of 2b protein in cytoplasm as well^27,28^. Phosphorylation site of 2b protein is conserved in all CMV isolates, KSPSE motifs proposed constitute motifs for casein kinase II (S/TxxD/E) and cyclin-dependent kinase 2 (40SP41) for phosphorylation. Deletion of the putative phosphorylation site (2bΔKSPSE) and two point mutations (2b40A, 2b42A) were analysed previously^27,29,30^, but the phosphorylated form of the 2b protein and the role of the actual phosphorylated state of the 2b protein were not analysed. To discern the impact of 2b protein phosphorylation, we examined the effect of mutations located in putative phosphorylation site in symptom induction, gene silencing suppressor activity as well as in subcellular localization.

## Results

### Phosphorylation of 2b protein *in vivo*

**Figure 1:**
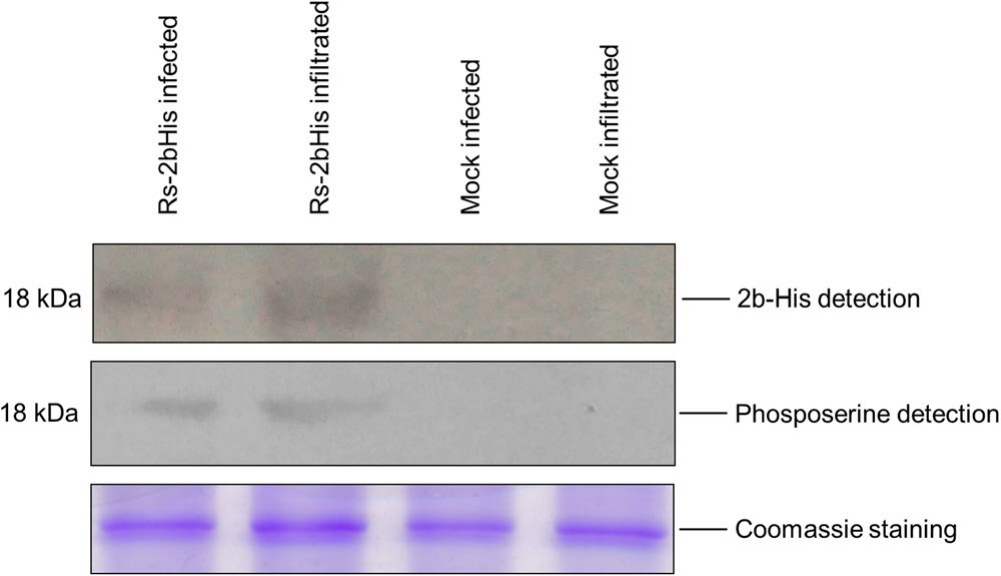
*In vivo* phosphorylation of *Cucumber mosaic virus* 2b protein. Western blot analysis of infected (10 dpi) and agroinfiltrated *N. benthamiana* plants with Rs-2bHis using penta-his and phosphoserine antibodies. Coomassie staining was used to monitor the equivalence of protein loading.

The putative phosphorylation site of CMV 2b protein (amino acids 39–43) located at the beginning of the second alpha helix is strictly conserved in both subgroups of the virus^26^. KSPSE has been proposed to constitute phosphorylation motifs for casein kinase II (S/TxxD/E) and cyclin-dependent kinase 2 (40SP41). To examine the *in vivo* phosphorylation state of the 2b protein, Western blot analysis was carried out by using phosphoserine antibodies. Since antibodies for 2b protein detection were not available, the coding region of histidine tagged 2b protein was cloned into the infectious cDNA clone of Rs-CMV RNA 2. It was previously found that attachment of a hexahistidine sequence did not alter the subcellular localization and the ability to bind sRNAs^23,28^. *Nicotiana benthamiana* plants were infected with the chimaera Rs-2bHis *in vitro* transcripts in the presence of the Rs-CMV RNA 1 and 3 transcripts. Parallel to the infection, *N. benthamiana* plants were infiltrated with the binary vector expressing the previously described 2bHis protein^23^. Ten days after inoculation (symptoms appeared on the upper non-inoculated leaves) and 4 days post infiltration, the accumulation and the phosphorylation of 2b protein were analysed by Western-blot using penta-his and phosphoserine antibodies (Fig. 1, Supplementary Fig. S1). The Western blot analysis proved the 2b protein phosphorylation *in vivo* in infected plant as well as in infiltrated patches (Fig. 1), but the mutant 2b/40-42/AAA was not phosphorylated in infiltrated patches (Fig. S1) demonstrating that the 40/42 amino acids are the exclusive phosphorylated amino acids of the 2b protein.

### Mutations in the 2b protein phosphorylation domain alter symptom induction of CMV

**Figure 2:**
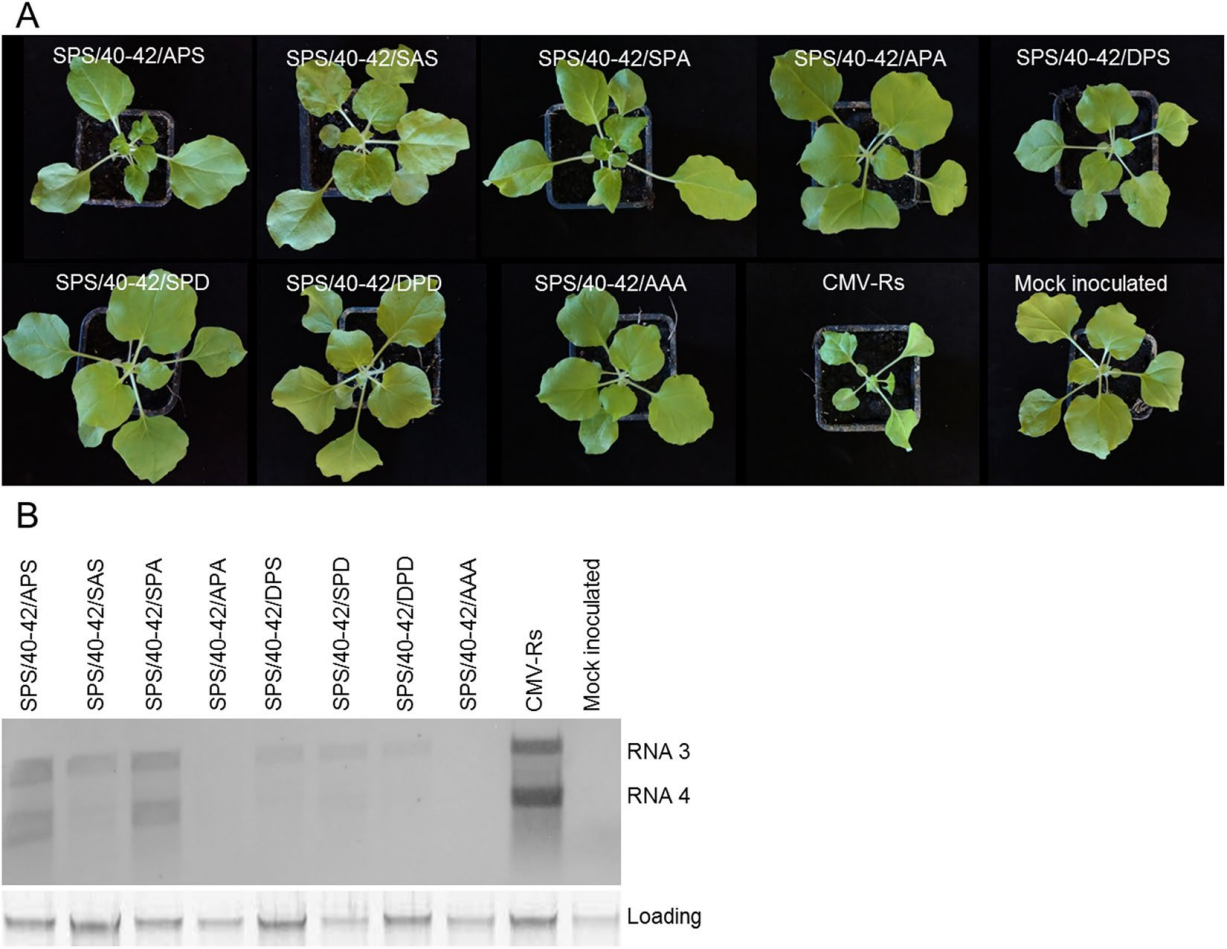
Symptoms (**A**) and accumulation of CMV and the different 2b protein mutants (**B**) in *N. benthamiana* plants. Infections with Rs-SPS/40-42/APA, Rs-SPS/40-42/DPD were asymptomatic during the monitored period (six weeks). Infection with Rs-SPS/40-42/SPD and Rs-SPS/40-42/DPS induced mild symptoms: mild leaf distortion, mild systemic mosaic, and mild stunting (**A**). Rs-SPS/40-42/APS, Rs-SPS/40-42/SAS and Rs-SPS/40-42/SPA exhibited severe symptoms, including systemic mosaic, leaf distortion, and chlorosis. However, symptoms induced by these mutants were never as severe as those induced by Rs-CMV. CMV accumulation in systemically infected leaves analysed by northern hybridization at 7 dpi. The viral RNA accumulation was in accordance with the strength of the symptoms. The radiolabelled probe was specific for Rs-CMV RNA 3. Ethidium bromide-stained rRNA from the same volume of the sample is shown below each lane.

In order to analyse the effect of the different phosphorylated state of 2b protein, seven different mutants were created. Substitutions were integrated into the Rs-CMV RNA 2 clone in the SPS motif for analysis of the effect of alanine (to mimic the non-phosphorylated state) and aspartic acid (to mimic the phosphorylated state) substitution in single or in both serine residues (40S, 42S). The substitution of serine amino acid with negatively charged aspartate residues, known to reproduce the electrostatic effect of phosphorylation as it was previously applied for example in the case of TMV MP30^31,32^. All of the mutations were integrated into the RNA 2 infectious clone of Rs-CMV resulting in the following constructs: 2b/40-42/SAS, 2b/40-42/APS, 2b/40-42/SPA, 2b/40-42/APA, 2b/40-42/DPS, 2b/40-42/SPD and 2b/40-42/DPD. To compare these mutants with wild-type virus (Rs-CMV) in terms of symptom induction and viral accumulation, plasmids containing the full-length clones of Rs-CMV RNAs were transcribed *in vitro* for inoculation of *N. benthamiana* plants. RNA 1 and 3 transcripts were combined with the wild-type RNA 2 and either with the mutated RNA 2 transcripts. The infection was followed by visual observation and northern blot analysis. Infections of *N. benthamiana* with Rs-SPS/40-42/APA and Rs-SPS/40-42/DPD were asymptomatic similarly to the previously described Rs-SPS/40-42/AAA^23^ during the monitored period (six weeks). Infection with Rs-SPS/40-42/SPD and Rs-SPS/40-42/DPS induced mild symptoms: mild leaf distortion, mild systemic mosaic, and mild stunting (Fig. 2A). *N. benthamiana* plants infected with Rs-SPS/40-42/APS, Rs-SPS/40-42/SAS and Rs-SPS/40-42/SPA exhibited severe symptoms, including systemic mosaic, leaf distortion, and chlorosis. However, symptoms induced by these mutants were never as severe as those induced by Rs-CMV in *N. benthamiana*. The northern analysis was in concordance with the symptom appearance (Fig. 2B). The identity of each of the mutants was verified by RT/PCR followed by nucleic acid sequence determination.

### Gene silencing suppressor activity of the mutant 2b proteins in patch assays

**Figure 3:**
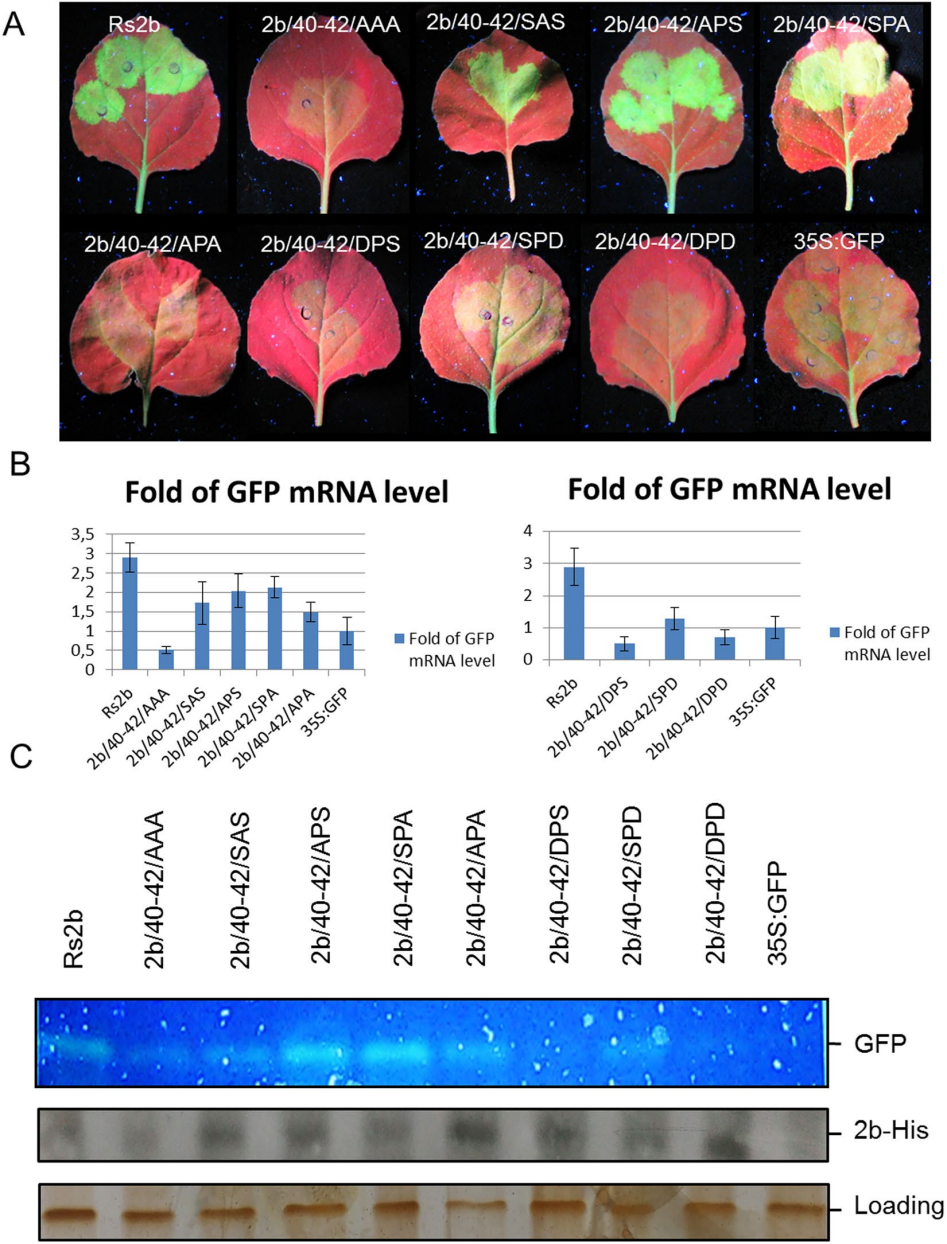
Analysis of suppression of transgene-induced silencing of Rs2b and the different mutants. A binary vector expressing the GFP reporter gene was co-infiltrated into *Nicotiana benthamiana* leaves with an empty binary vector or with binary vectors expressing 2b protein or 2b/40-42/AAA, 2b/40-42/APS, 2b/40-42/SPA, 2b/40-42/APA, 2b/40-42/SAS, 2b/40-42/DPS, 2b/40-42/SPD and 2b/40-42/DPS 2b protein construct (**A**). Mutants 2b/40-42/SAS, 2b/40-42/APS and 2b/40-42/SPA retained partial suppressor activity. Mutants 2b/40-42/DPS, 2b/40-42/SPD, 2b/40-42/DPD, 2b/40-42/APA and 2b/40-42/AAA failed to suppress GFP activity. The accumulation level of GFP RNA was quantitatively measured by qRT-PCR in the infiltrated leaves (**B**). Parallel detection of the fluorescence of GFP proteins on SDS-PAGE by illuminating the gel with UV lamp, immunoblot analyses of accumulation the His-tagged 2b protein mutants in agroinfiltrated patches by penta-his antibody detection and silver staining to monitor the equivalence of protein loading (**C**).

Gene silencing suppressor activity is the primary function of 2b protein, so this feature was analysed in *Agrobacterium*-mediated co-infiltration assay. Binary vector expressing GFP reporter gene was agroinfiltrated into transgenic *N. benthamiana* (silenced for GFP expression) leaves together with the binary vector expressing the wild type 2b protein or the different mutant ones (2b/40-42/AAA, 2b/40-42/SAS, 2b/40-42/APS, 2b/40-42/SPA, 2b/40-42/APA, 2b/40-42/DPS, 2b/40-42/SPD, 2b/40-42/DPD) (Fig. 3A). GFP silencing was visually observed at 4 days post agroinfiltration and the accumulation level of GFP RNA in the infiltrated leaves was quantitatively measured by qRT-PCR (Fig. 3B). We found that mutants 2b/40-42/SAS, 2b/40-42/APS and 2b/40-42/SPA retained partial suppressor activity. However, mutants 2b/40-42/DPS, 2b/40-42/SPD, 2b/40-42/DPD and 2b/40-42/APA failed to suppress GFP activity similarly to the previously described 2b/40-42/AAA^23^. The accumulation of the mutant proteins were analysed by western-blot using penta-his antibody to ascertain that the different GFP levels were caused by the different suppressor activities not by the instability of the proteins (Fig. 3C).

### Subcellular localization of 2b protein and its mutants monitored by BiFC

**Figure 4:**
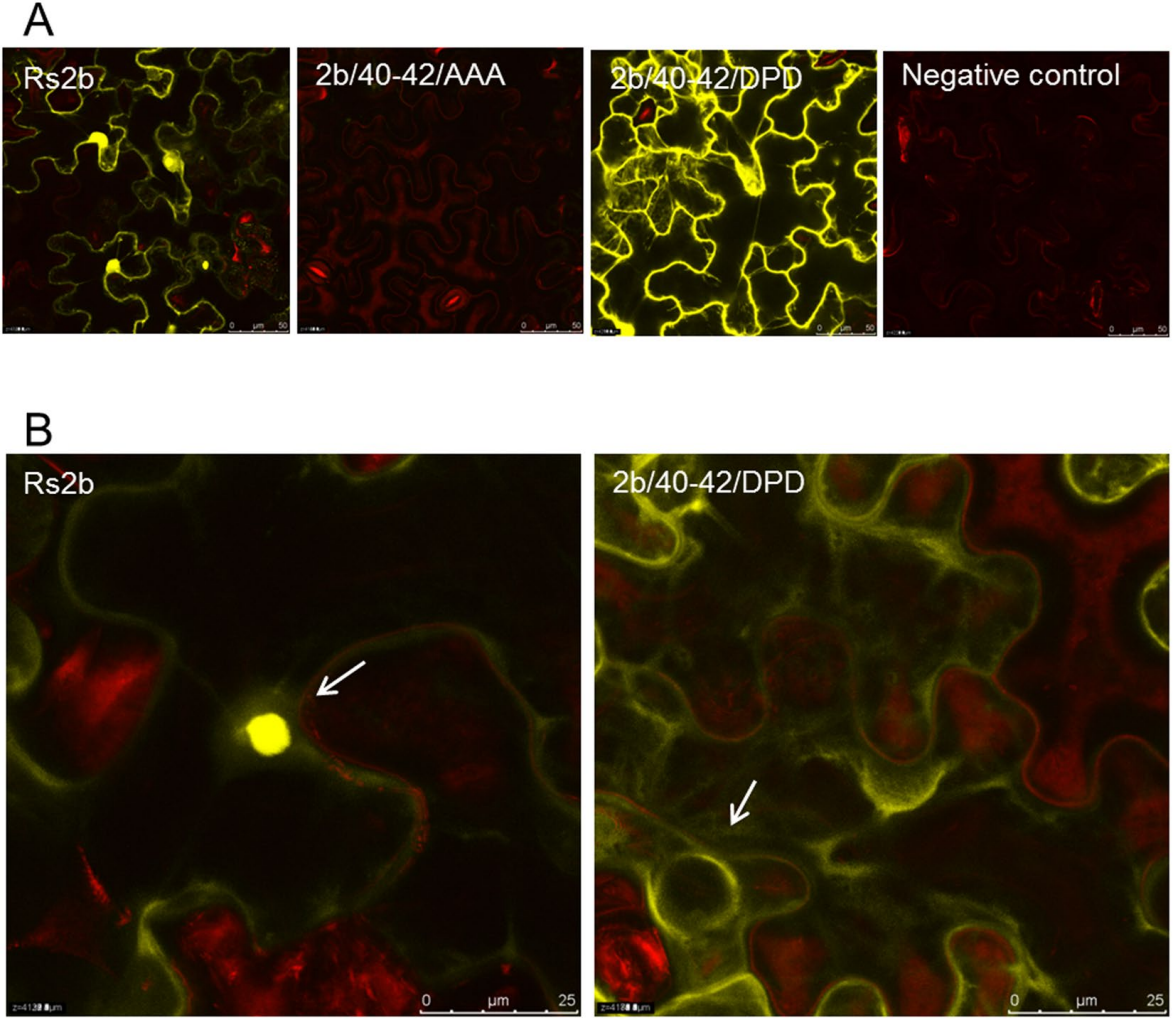
Subcellular localization of Rs-2b and its mutants detected by BiFC in *Nicotiana benthamiana* plants. *N. benthamiana* plants were co-agroinfiltrated with transiently expressed 2b proteins fused to either the N or C terminus of yellow fluorescent protein (YFP). Rs-CMV was clearly detectable in cytoplasm as well as in nuclei (**A**,**B**). No fluorescence was observed by confocal microscopic imaging in cells within leaf patches co-infiltrated with A. tumefaciens cells expressing 2b/40-42/AAA. In the case of mutant 2b/40-42/DPD fluorescence was detectable only in cytoplasm, not in the nucleus, nor in the nucleolus (**A**,**B**). The bar represents 50 µm (**A**) or 25 µm.

Previous studies suggest that beside nuclear localization signals the phosphorylation state of 2b protein affects its nuclear localization since deletion of the complete phosphorylation motif (2bΔKSPSE) altered the subcellular localization of 2b protein, confining it largely to the nucleolus^27^. To determine the impact of phosphorylation state of 2b protein we monitored the subcellular localization of the wild-type and mutated 2b proteins with bimolecular fluorescent complementation assay (BiFC). Previously, self-interaction of CMV 2b protein was demonstrated *in vivo* in *N. benthamiana* using BiFC^27^, and proposed that CMV 2b protein, like TAV (*Tomato aspermy virus*) 2b protein, functions in the form of a homodimer or higher order of oligomer as a suppressor of RNA silencing. Both cytoplasmic and nuclear localization of the dimers were demonstrated^27,28^. To analyse the subcellular localization of 2b protein *in vivo*, both the N-terminal and C-terminal fragments of YFP were fused to the C-terminus of 2b protein and also to the mutant 2b proteins with highly altered phenotype, generating fusion proteins (SPS/40-42/AAA and SPS/40-42/DPD). The 2b protein forms homodimers or higher oligomers therefore we used interaction of the wild-type 2b protein itself as a positive control applying BiFC in *N. benthamiana* plants. At 3 dpi, infiltrated leaves were subjected to confocal microscopy to monitor the reconstituted YFP signal. Strong YFP fluorescence in the cytoplasm and in the nucleus co-expressed Rs2b-N-YFPC and Rs2b-C-YFPC demonstrated that Rs-CMV was very apparent in cytoplasm as well as in nuclei, similarly to Gonzalez *et al*.^27^ (Fig. 4A). No fluorescence was observed by confocal microscopic imaging in cells within leaf patches co-infiltrated with *A. tumefaciens* cells expressing 2b/40-42/AAA. Interestingly, fluorescence was detectable only in cytoplasm in the case of mutant 2b/40-42/DPD, but fluorescence was not detected in the nucleus, or in the nucleolus (Fig. 4A,B). This observation indicates that the phosphorylated 2b protein accumulated in cytoplasm, phosphorylation of 2b protein prevents its accumulation in the nucleus.

### Subcellular localization of EGFP fused 2b protein and its mutants

**Figure 5:**
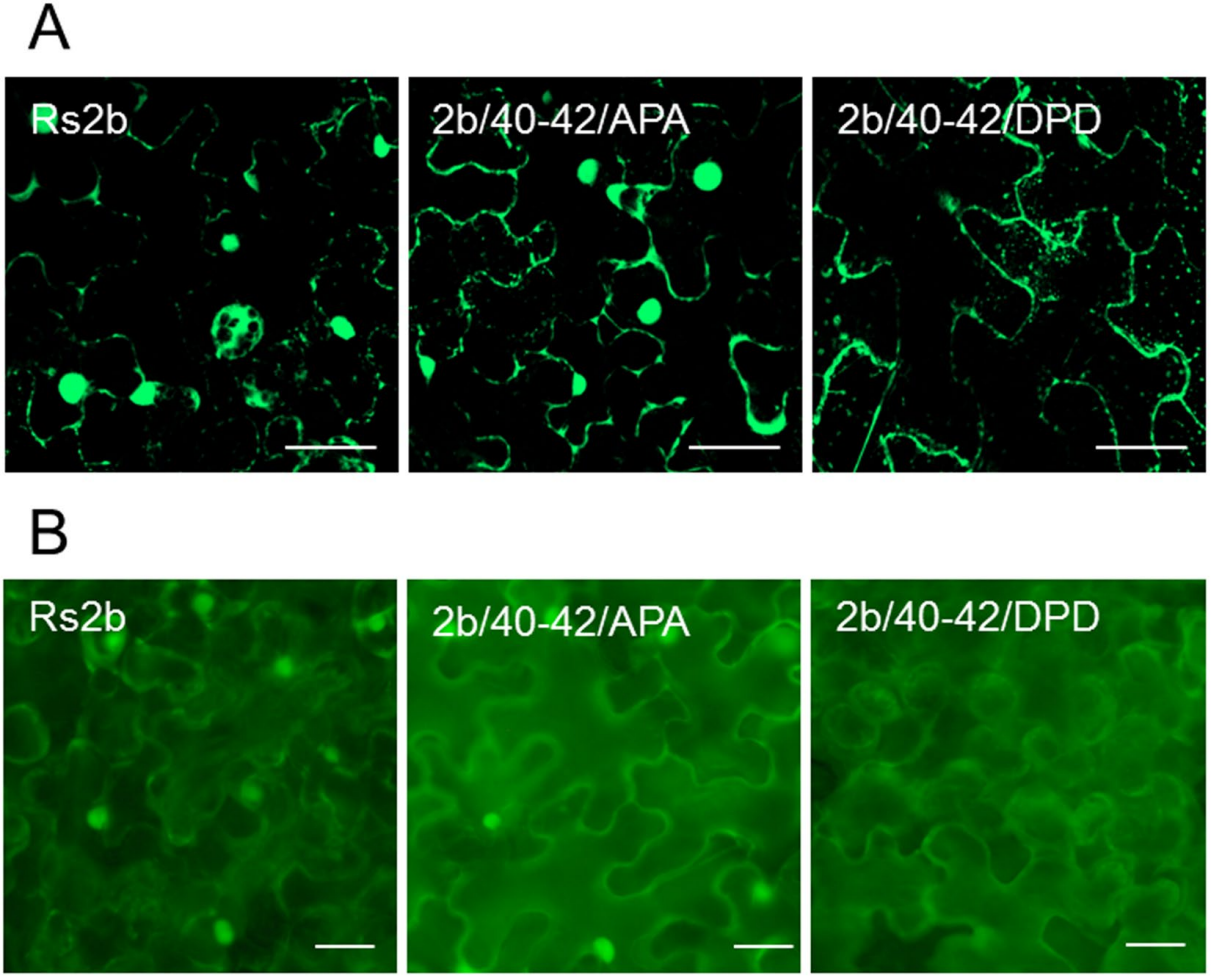
Subcellular localization of Rs2b-EGFP, 2b/40-42/APA-EGFP and 2b/40-42/DPD-EGFP in *N. benthamiana* plants. *N. benthamiana* plants were infiltrated with transiently expressed Rs2b-EGFP, 2b/40-42/APA-EGFP and 2b/40-42/DPD-EGFP fusion proteins. Rs2b-EGFP and 2b/40-42/APA-EGFP were clearly detectable in cytoplasm as well as in nuclei. The mutant 2b/40-42/DPD was detected in cytoplasm. The bar represents 50 µm.

The EGFP was fused to the C-terminus of the Rs2b protein and to the 2b/40-42/APA and 2b/40-42/DPD mutants in order to visualize the subcellular localization of the monomer form of the 2b protein as well. In infiltration experiments the Rs2b-EGFP and the 2b/40-42/APA-EGFP were detected in the cytoplasm and the nuclei, while the 2b/40-42/DPD-EGFP was detected only in the cytoplasm, but not in the nuclei (Fig. 5), confirming that the phosphorylated 2b protein is excluded from the nuclei.

### Nuclei isolation

**Figure 6:**
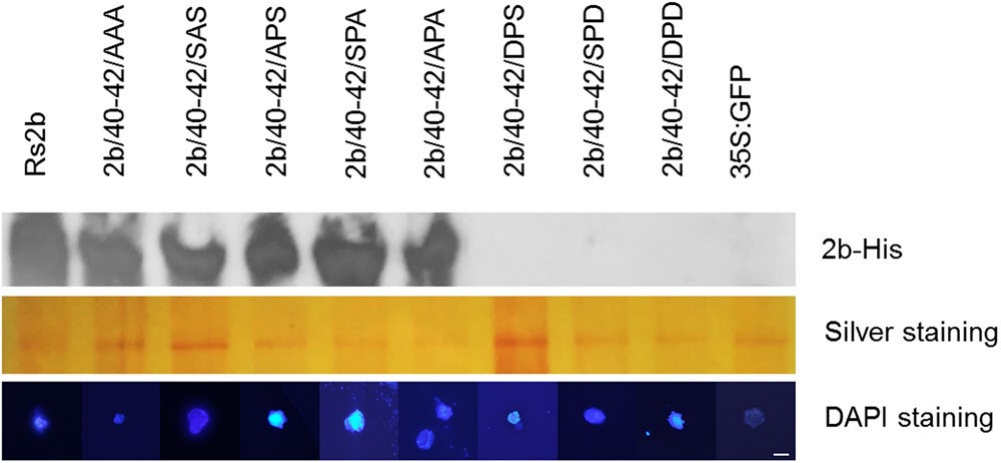
Western blot analysis of the Rs2b and the different mutants from cell nuclei purified from infiltrated *N. benthamiana* leaves. His-tagged 2b proteins were detected with penta-his antibody and silver staining was used to monitor the equivalence of protein loading. Mutants SPS/40-42/AAA, SPS/40-42/APA, and single mutants SPS/40-42/APS, SPS/40-42/SPA and SPS/40-42/SAS were detectable from purified nucleoli with penta-his antibody. The mutants 2b/40-42/DPS, 2b/40-42/SPD and 2b/40-42/DPD were not detectable in nuclei. DAPI-staining was used for visualisation of the purified nuclei (appearing blue) with fluorescence microscope using UV-light. The bar represents 10 µm.

To further analyse the subcellular localization of the mutants, nuclei were purified from agroinfiltrated leaf patches as described previously^33^. The presence of the constructs in the nuclei was verified by visual observation using microscopy as well as protein extraction and staining (Fig. 6). Mutants SPS/40-42/AAA, SPS/40-42/APA, and single mutants SPS/40-42/APS, SPS/40-42/SPA and SPS/40-42/SAS were detectable from purified nucleoli with penta-his antibody. The mutants 2b/40-42/DPS, 2b/40-42/SPD and 2b/40-42/DPD were not detectable in nuclei (Fig. 6). These findings further demonstrate that the phosphorylated state of 2b protein precludes it from accumulating in the nucleus and restricts the location of 2b protein exclusively to the cytoplasm.

## Discussion

**Figure 7:**
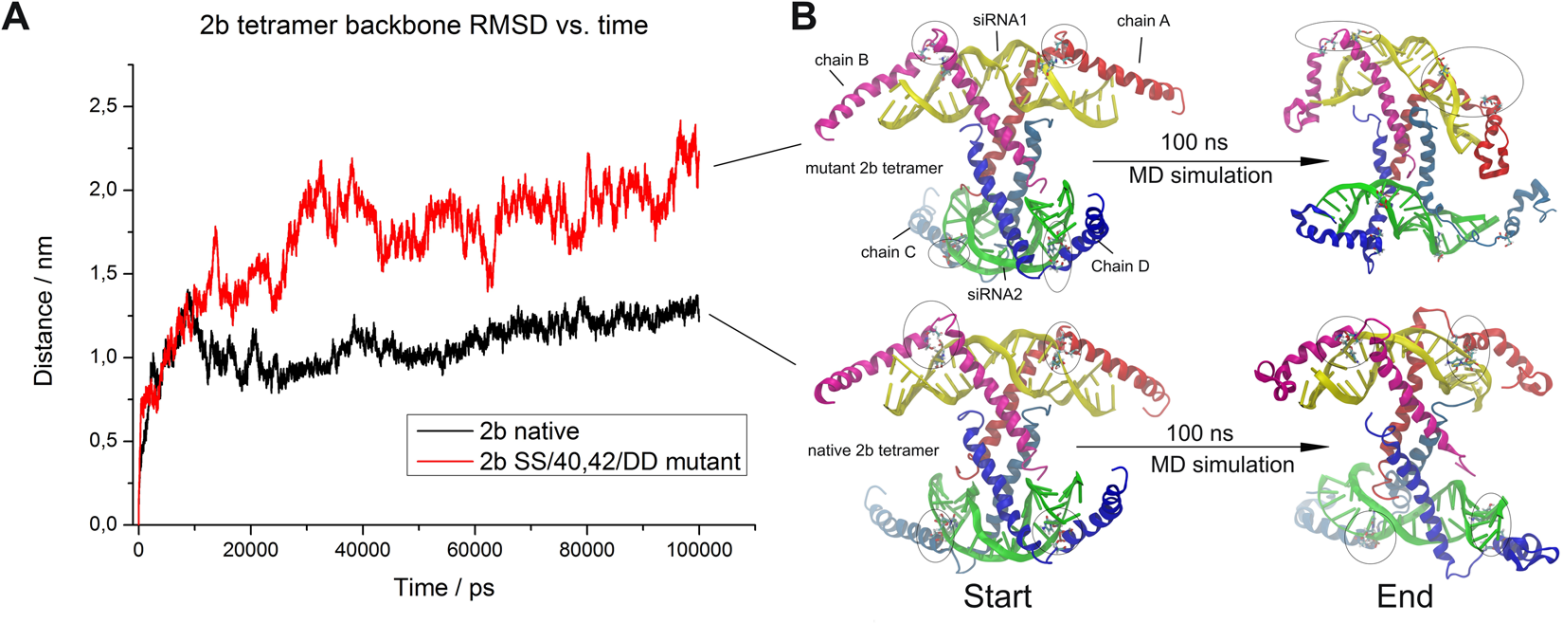
Analysis of the native and the mutant CMV 2b (SS/40,42/DD) tetramer MD simulations. The initial structures were taken as references when calculating RMSD values (**A**). The native siRNA-2b tetramer ribonucleoprotein complex reached a stable equilibrium state (black line), while the mutant complex became unstable (red line). Starting and ending structures of the MD simulation are in cartoon representation (**B**). The sites of residue 40 and 42 of the 2b chains are encircled.

**Figure 8:**
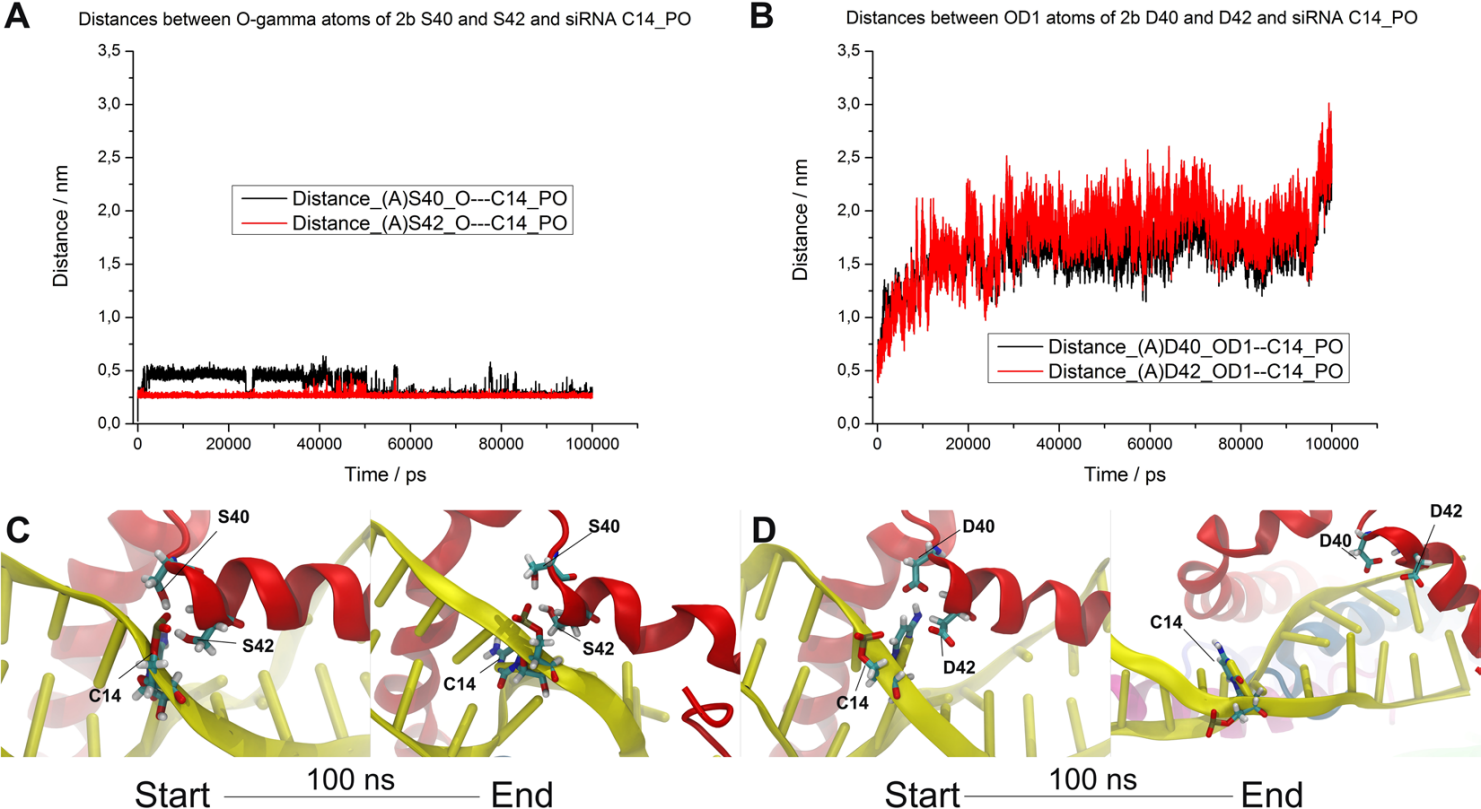
Residues 40 and 42 of the CMV 2b protein have key stabilizing role in the siRNA binding. The S40 and the S42 residues in the native 2b tetramer formed stable H-bonds with the phosphate oxygen atoms of the cytosine-14 (C14_PO) and/or guanosine-13 (C13_PO) (**A**,**C**). Sometimes, for only very short time these serine side chains swing toward the solvent. The D40 and the D42 residues in the mutant 2b tetramer disrupted the siRNA binding (**B**,**D**). CMV 2b protein chain A and the siRNA1 are in cartoon representation (**C**,**D**). The studied residues are in licorice representation.

Reversible phosphorylation is a well-studied posttranslational protein modification, which plays a fundamental role in the regulation of different protein activities *in vivo*. Several studies in case of different viruses suggest the physiological relevance of phosphorylation of movement protein^14,34^ and coat proteins in viral cell-to-cell movement^9,35^. Limited data is available about the function of CMV 2b protein phosphorylation. Previous sequence analyses have identified potential phosphorylation sites (amino acids 39–43) for casein kinase II (CKII) (S/TxxD/E) and CDK2 (40SP41), which are conserved in all of the cucumoviral 2b proteins, both in subgroup I and II as well as in TAV and in Peanut stunt virus (PSV)^26^. In this study, *in vivo* phosphorylation of 2b protein has been directly proved with transiently expressed 2b protein and in CMV infected *N. benthamiana* leaves (Fig. 1). Serine 40 and 42 located at the beginning of the second alpha-helix (Fig. 7B, encircled regions) was previously analysed with deletion mutants and site specific mutagenesis. It was previously described essential for nuclear accumulation and siRNAs binding to suppress PTGS^27,30^ as well as for symptom induction^29^. The deletion of the entire motif (mutant 2b/DKSPSE) abolished the suppressor activity of the protein reducing its ability to bind RNAs^27^. It is consonant with our results as mutant 2b/40-42/AAA failed to suppress GFP activity (Fig. 3). Interestingly, mutants 2b/40-42/SAS, 2b/40-42/APS, 2b/40-42/SPA and retained partial suppressor activity (Fig. 3). A CMV 2b homologue protein, TAV 2b forms a pair of hook-like dimers to recognize the siRNA duplex in a sequence independent and length-preference manner^36^. Studies using the TAV 2b homologue demonstrated that in aqueous solution its homodimers or oligomers are spontaneously formed, even in the absence of siRNAs^36^. Tetramer form is not necessary for siRNA binding and suppressor activity^27^. The examined mutations are located in the forepart of the second α-helix. The hydroxyl groups of serine 40 and 42 form H-bond(s) with the phosphate oxygen atoms of the cytosine-14 (C14_PO) and/or guanosine-13 (G13_PO) (Fig. 8A,C). These H-bonds give high stability to the whole 2b tetramer – siRNA complex (Fig. 7A,B). But, in the course of the MD simulation for only very short time periods (5–50 ps) these serine side chains swing toward the solvent where a host kinase can phosphorylate these serine residues. After the phosphorylation event the phospho-serine side chains cannot turn back to the negatively charged sugar-phosphate backbone of the siRNA, since their negative charges repel the 2b protein chain from the siRNA.

Beside phosphorylation domain, 2b protein also contains nuclear localization signals (NLS1 and NLS2)^27^. The molecular models of 2b dimers suggest that the NLS regions located at the first alpha-helix are involved not only in siRNA binding but act as interaction interface for importin protein, probably interaction with karyopherin α as suggested previously^25^. Our previous study also suggested that the less effective suppression of local gene silencing of NLS mutants is a result of the damaged structure and not the absence of nuclear localization^23^. It was also in accordance with a previous study by Gonzalez *et al*., as tagging GFP-2b with a NES resulted in the relocalization of the protein from the nucleus/nucleolus into the cytoplasm. The patch assays showed that NES-GFP-2b retained its ability to suppress RNA silencing^30^. The nuclear import of a subset of nuclear proteins that contain the classic nuclear localization signals is mediated by the importin α/β heterodimer (NLSs)^37–39^. Generally, α subunit specifically binds to basic NLSs in the cytoplasm, whereas the β subunit interacts with the NPC during the import process^39^. The nuclear import of 2b may rely on the importin-α/β transport system, and importin α could bind 2b by recognition of its nuclear localization signals (NLSs). Previously it was observed that a mutant with a deleted putative phosphorylation site was mainly localized to nucleoli, suggesting a role of phosphorylation in nuclear import^27^. Our results clearly demonstrate that mutants mimicking the non-phosphorylated state (SPS/40-42/AAA, SPS/40-42/APA, and single mutants SPS/40-42/APS, SPS/40-42/SPA and SPS/40-42/SAS) can be transported into the nucleus since they were detectable from purified nucleoli (Fig. 6). However, we could not detect mutants which mimicked phosphorylation (SPS/40-42/DPS, SPS/40-42/SPD and SPS/40-42/DPD) from nuclei. These results were also supported by the BiFC and 2b-EGFP fusion protein localization experiment as mutant SPS/40-42/DPD was localized in the cytoplasm but not in the nucleus (Figs 4 and 5). Besides *in vivo* experiments, dual phosphorylated state of 2b protein at S40 and S42 was also modelled *in silico*. The serine 40 and 42 were mutated into aspartate residues. Aspartate is a phosphomimetic of phospho-serine and can mimic the constant phosphorylated form of the protein. The initial conformation of the phosphomimetic form of the 2b tetramer – siRNA complex was similar to the native form. Nevertheless, due to the aforementioned negative – negative charge repulsion the ribonucleoprotein complex became very instable (Fig. 7). The distances between the mutated residues (D40, D42) and the phosphate oxygen atom of the siRNA C14-PO increased from the initial 0.25 nm to the range of 1 – 3 nm (Fig. 8B,D). However, during the 100 ns MD simulation the mutant 2b tetramer – siRNA complex did not completely come apart, but for example; the 2b (C, D chains) – siRNA2 dimer part were turned almost 180 degrees from the initial state (Fig. 7B). These findings are consistent with the patch assay results as mutants 2b/40-42/DPS, 2b/40-42/SPD, 2b/40-42/DPD failed to suppress GFP activity (Fig. 3).

In this study we identified a conserved dual phosphorylation switch in CMV 2b protein. Non-phosphorylated serine residues are essential in the 40 and 42 positions of the 2b protein to form stable ribonucleoprotein complex with double stranded siRNAs. Basically, two cases can be distinguished with regard to the 2b phosphorylation in relation to siRNA binding. First, if Ser-40 and Ser-42 of the monomer 2b protein is in phosphorylated state it cannot bind siRNA and obviously the ribonucleoprotein complex cannot be assembled. In the second case the stable 2b-siRNA complex (2b dimer with one double stranded siRNA or 2b tetramer with two double stranded siRNA) is phosphorylated by a host kinase and the ribonucleoprotein complex will disassemble. In accordance to these results, the impact of phosphorylation in the regulation of siRNA binding was demonstrated in the case of human Argonaute proteins, where RNA binding was strongly reduced in the phosphorylated state^40^.

Previously the nuclear-cytoplasmic partitioning of 2b protein was found determine the balance between its roles as a virulence determinant or as an RNA-silencing suppressor^28^. In this current study we have also compared the symptoms induced by the different 2b mutant viruses to the wild-type virus (Rs-CMV). Either point mutation of the two serine residues had effects on symptom induction since infection with the different mutants caused diminshed symptoms on *N. benthamiana* and led to a decreased levels of viral RNA compared to the wild-type CMV-Rs isolate (Fig. 2). Previous studies suggested that mutation of serine residue 40 or 42 reduces the ability of CMV to induce symptoms without impairing viral or 2b protein accumulation^29^. Single mutation in residue 41 (Pro41Ala) in TAV 2b protein showed a significant decrease in the local gene silencing suppressor activity^36^, similarly to the 2b protein of CMV, since we found that single mutation in residue 41 results in partial loss of suppressor activity (mutant SPS/40-42/SAS, Fig. 3).

Previously the role of phosphorylation in nuclear trafficking of proteins was demonstrated in several studies. For example in the case of human dUTPase, phosphorylation abolishes its nuclear import^41^. In the case of Epstein-Barr virus nuclear antigen, phosphorylation has a key role in the regulation of nuclear import^42^. Phosphorylation of nucleoprotein (NP) controls the nuclear-cytoplasmic shuttling of Influenza A virus^43^. More functional experiments will be needed to clearly identify the biological function of the phosphorylated 2b protein retained in the cytoplasm. Now it is conceivable that the phosphorylated 2b protein is a repository that can be easily converted into functional 2b protein available for suppressing the gene silencing process. Another possibility is sequestering the 2b protein for a different function, similarly to the Akt3-mediated phosphorylation of Ago2, that was proved to be a molecular switch between target mRNA cleavage and translational repression activities of Ago2^44^.

## Methods

### Plasmid constructions

Description of the Rs-CMV and the infectious transcripts (pRs1, pRs2, pRs3) has been published previously^45^. Mutant RNA 2 clones (2b/40-42/AAA, 2b/40-42/SAS, 2b/40-42/APS, 2b/40-42/SPA, 2b/40-42/APA, 2b/40-42/DPS, 2b/40-42/SPD, 2b/40-42/DPD) were generated by PCR based mutagenesis of pRs2 using the following forward primer 5′-CGAGATCTAATCTCAGACTGTTCCGCTTCC-3′. The sequences of reverse primers used are detailed in Supplementary Table S1.

DNA constructs encoding the Rs2b protein and its mutants (2b/40-42/APA and 2b/40-42/DPD) with EGFP fusion to C-terminus were created by overlapping PCR. The sequences of primers used for overlap PCR are detailed in Supplementary Table S1. Prior to *Agrobacterium* transformation, the identity of the contructs were verified by nucleotide sequence determination.

### 2b-His infectious clone

The 2b-His infectious clone was created from the previously described Rs2 GAF/108-110/AAA mutant and the pGEM-2bHis clones, which was generated in pRs2 plasmid DNA containing the full-length cDNA of CMV-Rs RNA 2^23^ as follows. The pGEM-2bHis clone was digested with *BamHI* (located downstream of the STOP codon) and blunt-ended with T4 DNA polymerase, and digested with *StuI* (at 2660 nt, in the middle of the 2b gene), and the insert was isolated. This fragment was ligated into the Rs2 GAF/108-110/AAA mutant digested with *PstI* and blunt ended with T4 DNA polymerase (at/prior the STOP codon) and *StuI* in the middle of the 2b gene. The resulting clone contained the complete 2b gene, the His-tag region and the STOP codon. The identity of pRs2b-His was verified by nucleotide sequence determination.

### Plant material, growth and inoculation

*Nicotiana benthamiana* plants were kept in environmentally controlled growth chambers at cycle of 14 h of light (23 °C) and 10 h of dark (18 °C). The plants were mechanically inoculated at the fourth-leaf stage with *in vitro* mutated RNA 2 transcripts in the presence of wild type RNA 1 and 3 transcripts or RNA 1, 2 and 3 of the wild type Rs-CMV transcripts. Symptom development was observed and recorded continuously during the following 2 weeks.

### Analysis of plants

Total RNA was extracted from 200 mg systemically infected leaves 7 days after inoculation^46^. Virus RNA accumulation was followed by northern blot analysis. Approximately 100 ng total RNA was denatured with formamide and separated in formaldehyde-containing agarose gels and blotted on to nylon membranes^47^. Northern blot hybridization analysis was performed with random-primed DIG-labeled DNA fragments specific for the Rs-CMV RNA3 sequence (Roche). RT-PCR/DNA sequence determination was performed to analyse the stability of the mutant virus. It was carried out with the Qiagen OneStep RT-PCR kit according to the manufacturer’s instructions, using primers flanking of 2b coding region (forward 5′-GTTTGCCTGGTGTTACGACACCGA-3′, reverse 5′-GCGGATCCTGGTCTCCTTTTGGAGGCCC-3′). PCR products were purified by High Pure PCR product Purification Kit (Roche) prior to nucleotide sequence determination.

### GFP imaging

For visual detection of GFP fluorescence patches on leaves and with PAGE, a Blak-Ray B-100SP UV lamp (UVP) was used, and images were taken with Canon PowerShot S5IS digital camera.

### Quantitative real-time RT-PCR

Fresh leaf tissues (30 mg) was ground in liquid N_2_ and extracted with SV Total RNA Isolation System (Promega). RNA concentration was measured by Nanodrop (Thermo, USA). Reverse transcription (RT) reaction was performed by RevertAid First Strand cDNA synthesis kit (Fermentas) according to the manufacturer’s instructions. All samples were run in triplicates. Primers 5′-AGTGGAGAGGGTGAAGGTGATG-3′ (forward) and 5′-TGATCTGGGTATCTTGAAAAGC-3′ (reverse) were used for GFP mRNA analysis. The *Nicotiana benthamiana* EF1 mRNA (GenBank accession number DQ321490) served as an internal control using primers 5′-TGGTGTCCTCAAGCCTGGTATGGTTG-3′ and 5′-ACGCTTGAGATCCTTAACCGCAACATTCTT-3′. Real-time PCR was carried out in BioRad CFX96 Touch™ machine, thermal cycling profile was described previously^23^.

### Construction of YFP fusion proteins for BiFC analysis

Agrobacterium-based binary vectors pGWBc-nYFP and pGWBc-cYFP for BiFC assay were constructed as described previously^48^. CMV 2b and mutant 2b ORFs were amplified by PCR using the following primers: 5′-GGGGACAAGTTTGTACAAAAAAGCAGGCTTCATGGAATTGAACGTAGGTGCAA-3′ and 5′-GGGGACCACTTTGTACAAGAAAGCTGGGTCGTGATGATGATGATGATGGAAAGCA-3′. All constructs were created by Gateway Technology (Invitrogen). The orientation of each insert was confirmed by sequencing.

### Agroinfiltration and confocal microscopy

Agrobacterium-mediated transient expression on *N. benthamiana* leaves was conducted by pressure infiltration as described previously^49^. Agrobacterium culture of p14-expressing strain was adjusted at 600 nm (OD_600_) to a final optical density of0.4 and the strains expressing the various 2b mutants to 0.2. The plants were maintained for 3 days at 22 °C in growth chamber (14 h light/10 h dark) before single discs of infiltrated *N. benthamiana* leaves were assayed for fluorescence by CLSM using a Leica TCS SP8 confocal microscope. The images were captured digitally and processed using the Leica confocal software.

To analyse the subcellular localization of the monomer Rs2b protein and its mutants, *N. benthamiana* plants were infiltrated with *A. tumefaciens* cell harboring plasmids pRs2b-EGFP, p2bAPA-EGFP and p2bPD-EGFP. *A. tumefaciens* cells were diluted to final optical density at 600 nm to 0.5. *N. benthamianna* plants were assayed 3 days after the infiltration using ZEISS LSM 410 Laser Scan Microscope and Olympus BH-2 Fluorescence Microscope. 488 nm laser was used for excitation and 525/50 barrier filter for GFP detection.

### Nuclei isolation

Agrobacterium-mediated transient expression on *Nicotiana benthamiana* leaves was conducted by pressure infiltration as described above. Agrobacterium culture of GFP-expressing strain was adjusted to a final optical density at 600 nm (OD_600_) 0.4 and the strains expressing the various 2b mutants to 0.2. Four days post-infiltration, nuclei were purified from agroinfiltrated leaf patches as described previously^33^. Thirty leaf discs from each 2b protein constructs were used for purification, nuclei were resuspended in 250 µl nuclei storage buffer. The presence of the purified nuclei of each construct has been verified by visual observation prior to DAPI staining (1 µl of nuclei in 7 µl of DAPI, 1 µg/ml) with an Olympus bx-51 microscopy using U-MNU2 filter, as well as by protein extraction and staining (1:1 with Laemmli buffer).

### Protein analysis, SDS-PAGE, and immunoblotting

Protein extracts from *N. benthamiana* leaves were prepared from leaf samples (20 mg, fresh weight). Leaf discs were ground and homogenized in an ice-cold mortar in Laemmli solution, heated at 95 °C for 5 min, and centrifuged (5 min at 10,000 g) to remove insoluble material. Aliquots of the supernatant (1 to 10 µL) were separated by SDS-PAGE on 20% gels. After electrophoresis, proteins were transferred to a Hybond-C membrane (GE Healthcare Bio-Sciences) and subjected to immunoblot analysis with Penta·His HRP Conjugate Kit following the manufacturer’s instructions (Qiagen). For phosphoserine detection we used Phosphoserine antibody (Qiagen), HRP conjugated mouse anti-Rabbit IgG as secondary antibody (Millipore) and for detection Pierce Western blotting substrate was used following the manufacturer’s instructions (Thermo Fischer Scientific).

### Modelling of 2b protein-siRNA complex

The three-dimensional structure of the CMV 2b was generated with I-TASSER^50^. The model was built using the Rs-CMV 2b sequence. The NCBI/GenBank accession number is AJ517801. The main template was the X-ray structure of TAV 2b (PDB ID code: 3CZ3) to create the alpha helical regions (aa 1–69). The biologically active tetramer form with siRNA duplexes (sequence of the 19nt siRNA is the same as in the 3CZ3 structure) was built with the Schrödinger Suite^51^ molecular modelling software package. The SPS/40-42/DPD mutant complex was derived from the native model using the “mutate residue” option of the molecular modelling software. The completed tetramer-siRNA ribonucleoprotein complexes were refined with energy minimization to eliminate the steric conflicts between the protein and RNA atoms.

### Molecular dynamics and graphics

Prior to MD simulations the Desmond system building panel (incorporated into Schrödinger Suite) was used to construct the solvated siRNA-2b tetramer ribonucleoprotein complexes. 0.15 M NaCl salt concentrations were used in both simulations. The OPLS-AA/2005 force field was used. Before starting the 100 nanosecond production runs the systems were prepared with energy minimizations and short restrained MD simulations: first, only the position of the solvent molecules was minimized and then the potential energy of the whole system without any restraints. In the subsequent step on an NVT ensemble, a short 12 ps position-restrained MD simulation at 10 K was carried out using a Berendsen thermostat to ensure the proper solute settlement around the protein. After this, 12 ps of position restrained MD simulation was performed on an NPT ensemble at 10 K and 1 bar using a Berendsen thermostat and pressure coupling. Then a 12 ps restrained NPT MD simulation warmed the solute to 300 K at 1 bar. Finally, a 24 ps non restrained NPT MD simulation prepared the systems for the production run at 300 K and 1 bar.

The MD simulations were initiated on the NPT ensemble 300 K and 1 bar using the Desmond^52^ molecular dynamics software application. The simulations were evaluated by the Desmond “simulation event analysis” application package of the Schrödinger Suite. The computing capacities were around 4-6 ns/day for the 2b tetramer-siRNA ribonucleoprotein complex simulations. The MD simulations were performed on the MTA Cloud supercomputing centre using two virtual computers (instances) with 16 VCPUs, respectively. The VMD version 1.9.3^53^ was used to create spectacular molecular graphics.

## Electronic supplementary material

Supplementary Table S1

Supplementary Figure S1
