# Supplementary material for: Phosphorylation regulates the subcellular localization of Cucumber Mosaic Virus 2b protein
Source: Sci Rep. 2017 Oct 18;7:13444. doi: 10.1038/s41598-017-13870-7 (PMC5647415; doi:10.1038/s41598-017-13870-7)
Supplement: Supplementary file 1 — Supplementary Table S1 [file 41598_2017_13870_MOESM1_ESM.pdf]

Phosphorylation regulates the subcellular localization of Cucumber Mosaic Virus 2b protein

Katalin Nemes[1], Ákos Gellért[2], Asztéria Almási[1], Pál Vági[1,3], Réka Sáray[1], Katalin Kádár[1], Katalin Salánki[1*]

Mutation	Primer sequences 5'-3'
SPS/40-42/APS	CCAGATCTCGCTCTCTCGCTGGGAGCTTTGTGACCTCGTTCCCG
SPS/40-42/DPS	CCAGATCTCGCTCTCTCGCTGGGCTATTTGTGACCTCGTTCCCG
SPS/40-42/SPA	CCAGATCTCGCTCTCTCGGCGGGACTTTTGTGACCTCGTTCCCG
SPS/40-42/SPD	CCAGATCTCGCTCTCTCGTCGGGACTTTTGTGACCTCGTTCCCG
SPS/40-42/DPD	CCAGATCTCGCTCTCTCGTCGGGCTATTTGTGACCTCGTTCCCG
SPS/40-42/APA	CCAGATCTCGCTCTCTCGGCGGGAGCTTTGTGACCTCGTTCCCG
SPS/40-42/SAS	CCAGATCTCGCTCTCTCGCTGGCACTTTTGTGACCTCGTTCCCG
RsRNA4Afor	ATTGAGCTCGTAGTACAGAGTTCAGGG
2bGFPrev	GCTCCTCGCCCTTGCTCACCATGAAAGCACCTTCCGCCCATTCGTTAC
2bGFPfor	GTAACGAATGGGCGGAAGGTGCTTTCATGGTGAGCAAGGGCGAGGAGC
GFPSTOP3'noncodingfor	CTCGGCATGGACGAGCTGTACAAGTGAAACCTCCCCTTCCGCATCTCC
GFPSTOPnoncodingrev	GGAGATGCGGAAGGGGAGGTTTCACTTGTACAGCTCGTCCATGCCGAG
CMVuni3'REV	GCGGATCCTGGTCTCCTTTTGGAGGCCC

Supplementary Table S1. The sequences of reverse primers used for creating mutations into the phosphorylation site of CMV 2b protein and primers used for creating EGFP fused 2b protein and mutants with overlap-PCR.
