# Supplementary material for: Phosphorylation regulates the subcellular localization of Cucumber Mosaic Virus 2b protein
Source: Sci Rep. 2017 Oct 18;7:13444. doi: 10.1038/s41598-017-13870-7 (PMC5647415; doi:10.1038/s41598-017-13870-7)
Supplement: Supplementary file 2 — Supplementary Figure S1 [file 41598_2017_13870_MOESM2_ESM.pdf]

Phosphorylation regulates the subcellular localization of Cucumber Mosaic Virus 2b protein

Katalin Nemes[1], Ákos Gellért[2], Asztéria Almási[1], Pál Vági[1,3], Réka Sáray[1], Katalin Kádár[1], Katalin Salánki[1*]

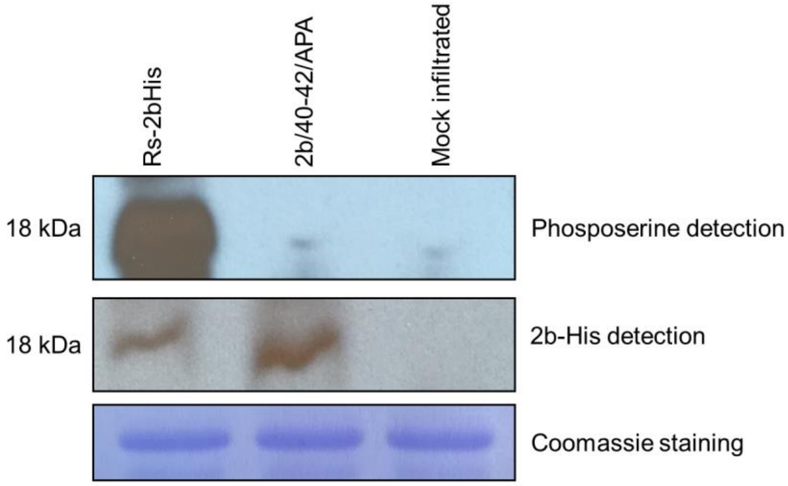

Supplementary Figure S1. *In vivo* phosphorylation of Cucumber mosaic virus 2b protein and mutant 2b/40-42/APA in agroinfiltrated *N. benthamiana* plants. Western blot analysis of agroinfiltrated *N. benthamiana* plants with Rs2b-His and 2b/40-42/APA-His using phoshoserine and penta-his antibodies. The equal loading of the proteins was detected by Coomassie staining.
